# Group exercise interventions and their impact on patient-reported outcomes in urologic, gynecologic, and breast cancers

**DOI:** 10.1007/s00520-026-11447-7

**Published:** 2026-11-12

**Authors:** Christine Ibilibor, Andrea H. Denton, Shannon D. Armbruster

**Affiliations:** 1 Department of Urology, Division of Urologic Oncology, University of Virginia, Charlottesville, VA USA; 2 Claude Moore Health Sciences Library, University of Virginia, Charlottesville, VA USA; 3 Department of Gynecologic Oncology, Carilion Clinic, Roanoke, VA USA

**Keywords:** Group, Physical exercise, Quality of life, Urology, Gynecology, Breast cancer

## Abstract

**Background/objective:**

Patients with urologic, gynecologic, and breast malignancies experience unique treatment-related side effects that can be socially isolating. While group-based exercise can provide social support, its impact on these patients remains unclear. This review aims to examine patient-reported outcomes related to participation in group exercise in these patients.

**Methods:**

We performed a systematic review of randomized control trials and prospective cohort studies that compared group-based exercise to usual care or individual exercise in patients with a urologic, gynecologic, or breast malignancy and examined patient-reported metrics. PubMed/MEDLINE, Embase, CINAHL, SPORTDiscus, Web of Science, and Cochrane Database of Systematic Reviews were searched between January 2005 and December 2025.

**Results:**

We identified 973 studies and 14 met inclusion criteria. Of the included articles, 9 included breast, 4 included prostate, and 1 included bladder cancer patients, representing 1128 patients. Group exercise was performed in supervised groups or teams of 3–14 participants in a community or hospital facility. Compared to individual exercise, a group exercise intervention showed an improvement in patient-reported quality of life in 1 study where group dynamics were measured.

**Conclusion:**

This review was unable to show an advantage in group-based exercise compared to individual exercise with regard to patient-reported quality of life for patients with a breast, prostate, or bladder cancer. However, our study is limited by the absence of measured positive group dynamic in each study. Future investigations will include qualitative and quantitative measures of group dynamic and group cohesion to determine the potentially socially supportive role of group-based exercise interventions.

**Supplementary Information:**

The online version contains supplementary material available at https://doi.org/10.1007/s00520-026-11447-7.

## Introduction

Patients with urologic, gynecologic, and breast cancers can experience symptoms of urinary and sexual dysfunction as a consequence of their malignancy. In addition, the treatment of malignancies of the genitourinary and gynecologic tracts can negatively impact self-esteem and social functioning. A study by Kinnaird et al. showed that 57% of prostate cancer patients experience sexual dysfunction that resulted in new onset low self-esteem after treatment of their disease [1]. In addition, patients with cervical and prostate cancer treated with radiation therapy have reported a withdrawal from their normal activities as a result of treatment-related bowel dysfunction [2]. Similarly, patients with bladder cancer treated with radiotherapy reported distress due to diarrhea and fecal urgency post-treatment [3]. Endometrial cancer management can be associated with an increased likelihood of bowel dysfunction which has negative psychosocial implications [2]. In addition, 95–75% of patients with breast cancer have reported poorer body image as a result of their cancer treatment [2, 4, 5]. Thus, patients with these malignancies may experience treatment-related symptoms that can negatively impact self-image and contribute to social isolation, making social support an important mitigating factor [6].

Exercise remains an important adjunct to cancer survivorship and treatment because it can help alleviate treatment-related side effects in urologic, gynecologic, and breast cancers [7–9]. For example, a recent randomized controlled trial showed that patients with metastatic breast cancer who participated in a 9-month resistance and aerobic exercise program experienced a clinically meaningful increase in their sexual functioning at 6 months on quality-of-life questionnaires compared to control [10]. In addition, a prospective cohort study in prostate cancer showed that men who engaged in exercise prior to surgery experienced improved self-reported sexual function [11]. Thus, group exercise serves as a possible mechanism for providing both improved patient-reported outcomes and psychosocial support for this cancer population through peer support and shared experiences [12–14]. However, data investigating the impact of group exercise on these patient-reported metrics are sparse. Thus, this review serves to fill this gap.

## Materials and methods

This review comparing the effects of group-based exercise to individual exercise or usual care in patients with a urologic, gynecologic, or breast cancer on patient-reported outcomes was registered a priori as a protocol in PROSPERO (CRD420251185911) and was reported according to the Preferred Reporting Items for Systematic Reviews and Meta-Analysis (PRISMA) guidelines [15].

### Eligibility criteria

We reviewed the use of group-based physical exercise interventions in patients with a urologic (i.e., prostate, bladder, kidney, testicular, penile), gynecologic (i.e., ovarian, endometrial, cervical, vulvar, vaginal), or breast cancer compared to individual exercise or usual care in randomized clinical trials and comparative prospective cohort studies. Participants could receive active treatment for their malignancy during the intervention period or have completed their respective management strategy. Group-based exercise was defined as any physical activity as determined by the study authors performed among participants in a dyad or as a group of 3 or more participants. Participants could perform group exercise supervised, unsupervised, at a facility, in the community, or at home. Studies were excluded if group exercise was not compared to individual exercise, usual care, or if there was no control arm. Studies were also excluded if there was no evaluation of the impact of group exercise on patient-reported outcomes relating to quality of life, fatigue, sexual function, depression, anxiety, psychological well-being, or psychosocial support. Qualitative studies and randomized controlled trial protocols about group exercise were excluded.

### Intervention

Eligible studies investigated physical exercise in a dyad, team, or a group of 3 or more participants, with opportunities for interpersonal interactions between participants either in-person or through virtual platforms. Group exercise could include strength training, endurance training, stretching, breathing exercises, or aerobic exercise of any intensity performed in combination with nutritional support, psychosocial support, or educational interventions.

### Data sources

PubMed/MEDLINE, Embase, CINAHL, SPORTDiscus, Web of Science, and Cochrane Database of Systematic Reviews were searched by a medical librarian (A.H.D.) in December 2025 and limited to studies published since January 2005. Lists of keywords and phrases were created for both main concepts: (1) group exercise and (2) urologic cancers, gynecologic cancers, and breast cancer. Customized search strategies were developed for each database, utilizing keywords identified above as well as controlled vocabularies (e.g., Medical Subject Headings). Search results were limited to English language. The complete search strategies are listed in the Supplemental Text.

### Data extraction

Search results were imported into Covidence for de-duplication and review [16]. A 2-step screening method was utilized such that two authors (C.I. and S.D.A.) independently screened the title and abstract of all citations for inclusion, then included articles were read in full to confirm eligibility. Discrepancies were resolved through discussion between the two authors. Study characteristics, baseline participant demographics, clinical and pathologic cancer characteristics, group exercise program details, and patient-reported outcome data were collected using a form adapted from the Cochrane Collaboration’s data extraction template [17]. The risk of bias in the methodologies for each included study was determined, using the Cochrane Risk of Bias Tool [18]. Each included article was given a designation of low, high, or some concern for bias based on their randomization process, allocation concealment, deviation from the intended intervention, presence of missing data, selection of the reported outcome, and outcome measurement.

### Data synthesis

The primary outcome for this systematic review was change from baseline in patient-reported quality-of-life questionnaire scores after a group-exercise intervention. Secondary endpoints were changes in any patient-reported outcome measure after group exercise including patient-reported fatigue, sexual function, depression, anxiety, psychological well-being, or psychosocial support. These outcomes were chosen given the known positive impact of group-based peer support on these measures through enhanced coping, shared experiences, and general camaraderie [12–14]. Other secondary outcomes included changes in metrics of physical fitness and activity level defined by individual studies. To examine the potential effect of group exercise on our primary and secondary outcomes, we summarized any negative or positive associations with descriptive statistics.

## Results

**Fig. 1:**
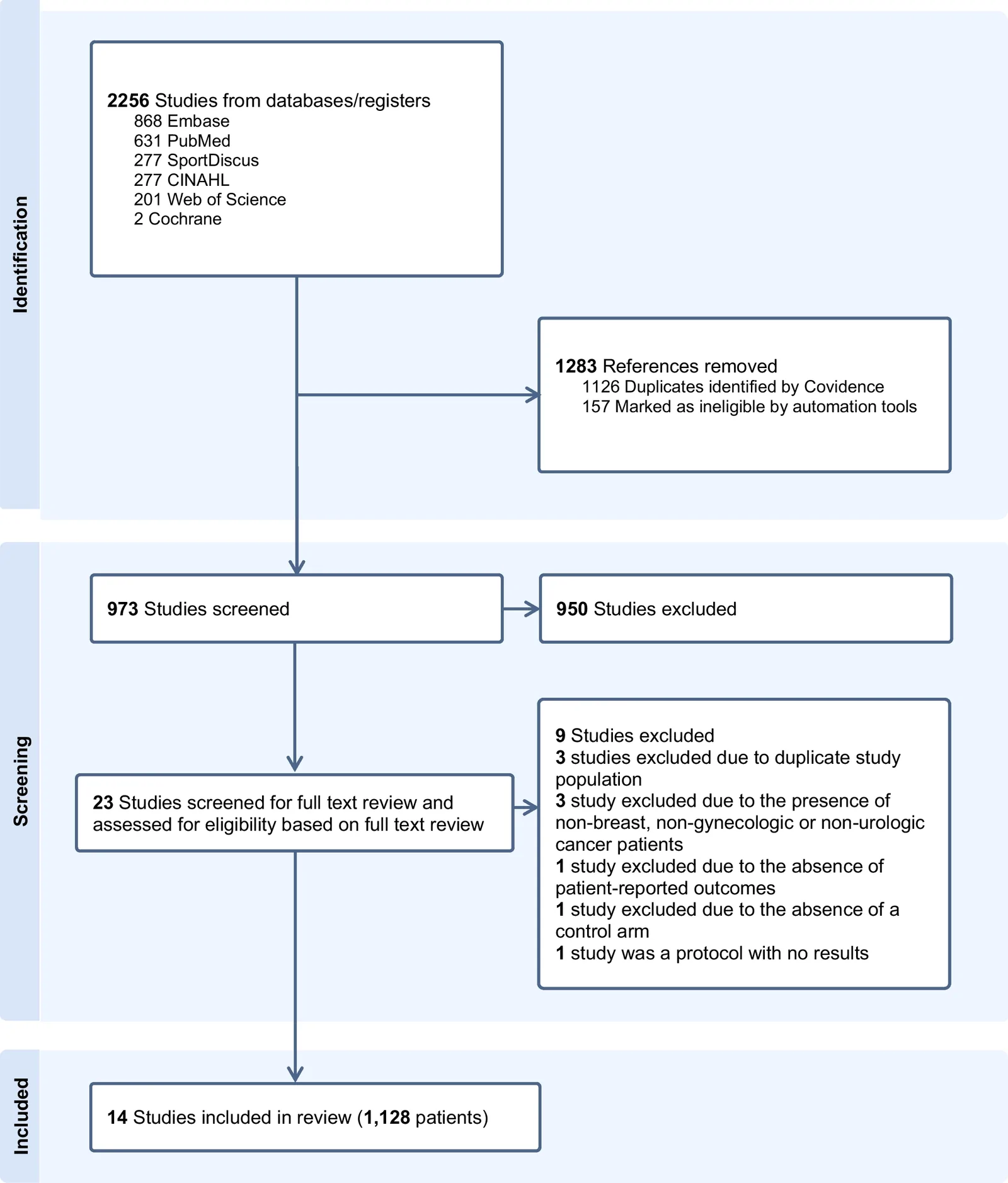
PRISMA flow diagram

Our search retrieved 973 citations, the full text of 23 studies was reviewed, and 14 were eligible for inclusion in the final analysis [19–32]. The PRISMA flow diagram is depicted in Fig. 1. During the full text review, the two most common reasons for article exclusion were the presence of duplicate study population (3 studies) and the presence of non-breast, non-gynecologic or non-urologic cancer patients (3 studies). The 14 studies represent 1128 patients of whom 679 (60%) were women, 449 (40%) were men, wherein 571 (51%) received a group-based exercise intervention, 370 (33%) received usual care, and 187 (17%) received an individual-based exercise intervention [19, 20, 22–32]. The approximate mean age was 58.5 years in the group-based exercise arm and 58.9 years in the control arm. Race or ethnicity was reported in only 1 article [19]. Included patients were from different countries with Australia (2 studies), Canada (2 studies), Denmark (2 studies), South Korea (2 studies), and the USA (2 studies) representing 70% (789 patients) of the sample population. The sample population included primarily breast (9 studies) and prostate cancer patients (4 studies) while only 1 study included bladder cancer patients. Eight studies included patients with early-stage cancer while the rest (6 studies) included both early and late-stage disease.

Eleven of the studies were randomized controlled trials (RCT), and 3 were comparative prospective cohort studies. Among the RCTs, 7 studies included a usual care control arm, whereas 4 used an individual-based exercise comparator. Of the prospective cohort studies, 1 included a usual care control arm and 2 utilized an individual-based exercise comparator (Table 1).

**Table 1:** Baseline study characteristics

Study	Country	Start year	Follow-up (mo)	Study size (n)		Design	Mean age, years (SD)		Mean BMI (SD)		Cancer type	Tumor stage (early or late)	Cancer treatment setting	Type of control arm	Primary outcome findings
				GRP	CTR		GRP	CTR	GRP	CTR					
Alibhai et al. (2024) [19]	Canada	2016	6	20	18	2-arm RCT	69.6 (9.8)	71.2 (8.3)	27.5 (3.6)	30.7 (4.9)	Prostate	Early and late	Receiving ADT	Unsupervised individual home-based aerobic and resistance exercise 4–5 days per week facilitated by health coaching	Individual home-based exercise was not inferior to group-based exercise with regard to its impact on physical endurance and fatigue based on the co-primary outcomes (FACT-F and 6MWT)
Bjerre et al. (2019) [20]	Denmark	2015	12	109	105	2-arm RCT	67.8 (6.2)	69 (6.2)	NR	NR	Prostate	Early and late	Prior prostatectomy, radiation alone, radiation with ADT, or chemotherapy	Usual care	Club-based football was associated with better bone mineral density and mental health based on the Short Form 12 compared to usual care
Borsati et al. (2024) [21]	Italy	2019	3	4	43	3-arm PrCH	55.6 (14.6)	54.9 (8.1)–53.3 (9)	31 (3.6)	27.7 (6.2)–26.7 (6.5)	Breast	Early and late	Prior surgery, radiation, target therapy, chemotherapy, or hormone therapy	Supervised individual facility-based or unsupervised individual home-based aerobic and resistance exercise	A patient-preferred exercise in the form of either group or individual exercise is feasible for women with breast cancer
Cho et al. (2006) [22]	South Korea	2002	2.5	27	28	2-arm RCT	48.7 (9.1)	49.6 (6.2)	NR	NR	Breast	Early	Prior chemotherapy and/or radiation with or without hormone therapy after mastectomy	Usual care	The group rehabilitation program improved shoulder range of motion, psychosocial adjustment, and quality of life compared to usual care
Desbiens et al. (2017) [23]	Canada	2013	3	12	14	2-arm RCT	70.7*	67.5*	28.8*	25.6*	Breast	Early	Receiving Aromatase inhibitor treatment after radiation with or without chemotherapy	Unsupervised individual video-assisted aerobic and strength training	Low recruitment prevented appropriate statistical comparison between group and individual exercise; however, both strategies showed improvement in fatigue scores and physical capacity
Galvao et al. (2025) [24]	Australia	2014	6	75	37	3-arm RCT	66.5 (6.7)–64.6 (7.9	67.8 (6.5)	28.4 (3.9)–27.6 (3.7	29.0 (5.0)	Prostate	Early and late	Prior prostatectomy, radiation, brachytherapy, receiving ADT, or other treatments	Usual care	The group exercise intervention with or without the psychosocial education was associated with improved sexual function based on IIEF scores compared to usual care
Kim et al. (2020) [25]	South Korea	2017	12	23	25	2-arm RCT	49.91 (7.62)	48.48 (6.75)	NR	NR	Breast	Early	Prior mastectomy, or lumpectomy with chemotherapy with or without radiation, or targeted therapy	Usual care	Group-based exercise resulted in an improvement in fatigue, depression, and sleep quality that was not different compared to usual care
Leach et al. (2019) [26]	USA	2016	2	14	12	2-arm RCT	51.8 (9.2)	51.9 (8.3)	29 (7)	29.6 (8.1)	Breast	Early	Prior surgery, radiation, or chemotherapy	Individual personal training	Group-based exercise that integrates group cohesion and group dynamics strategies may improve quality of life more than individual exercise training
Mardani et al. (2020) [27]	Iran	2017	2.5	35	36	2-arm RCT	69.40 (5.77)	70.39 (5.45)	24.40 (2.77)	24.64 (2.43)	Prostate	Early	Prior surgery, radiation, or hormone therapy	Usual care	Exercise improved quality of life in the domains of physical function, role function, and fatigue in prostate cancer patients compared to usual care
Møller et al. (2020) [28]	Denmark	NR	9.75	75	78	2-arm RCT	51.5 (9.6)	52 (9.3)	26.2 (5.3)	26 (4.9)	Breast	Early	Receiving adjuvant chemotherapy	12-week home-based individual pedometer tracking with health counselling and symptom guidance	Exercise interventions in the form of either group or individual exercise are able to mitigate the decline in cardiorespiratory fitness associated with chemotherapy among physically inactive breast cancer patients
Mutrie et al. (2007) [29]	Scotland	2004	6	101	102	2-arm RCT	51.3 (10.3)	51.8 (8.7)	27.3 (5.2)	27.5 (6.0)	Breast	Early	Mastectomy, lumpectomy, reconstructive surgery, radiation, chemotherapy, or combination	Usual care	Group-based exercise had no significant effect on general quality of life at 3 in 6 months compared to usual care. However, a positive effect on breast cancer-specific quality of life was seen at 6 months compared to usual care
Naumann et al. (2012) [30]	Australia	NR	2.25	14	22	3-arm PrCH	57 (9.5)	49 (7.9)–54.5 (9.5)	NR	NR	Breast	Early	NR	Supervised 9-week individual-based exercise and counselling or usual care	Group-based and individual-based exercise interventions with counselling are feasible for the breast cancer population
Porserud et al. (2014) [31]	Sweden	2008	12	9	9	2-arm RCT	72 (5)	72 (4)	23 (3)	25 (3)	Bladder	Early and late	Radical cystectomy	Usual care	Group exercise was able to improve distance walked compared to usual care up to a year post-operatively in bladder cancer patients but the hospital setting for the group exercise intervention was not feasible due to low recruitment
Tjoe et al. (2020) [32]	USA	2013	3.5	53	28	2-arm PrCH	51 (7)	56 (10)	29.5 (6.2)	31.5 (8)	Breast	Early and late	Surgery with/without adjuvant chemotherapy, surgery with/without neoadjuvant chemotherapy, or adjuvant radiation	Usual care	Team-based triathlon exercise training improved quality-of-life scores and endurance in breast cancer patients compared to usual care

*Only median age and BMI were reported

The risk of bias among the included studies was low since most studies were randomized, provided clear information on how outcome variables were measured and reported. Though most studies exhibited some bias in the effect of assignment to intervention domain because the majority of studies did not perform an intention-to-treat analysis nor a per-protocol analysis (9 studies). In addition, many of the included studies did not report on the rate of deviation from the prescribed intervention (8 studies). Due to the nature of group exercise interventions, personnel delivering the intervention were not blinded to the group allocation in 11 studies (Supplemental Table 1).

### Group exercise program structure

The group-based exercise intervention in the included studies involved aerobic exercise combined with resistance or strength training in groups of 3–14 participants per session. Sessions were generally low to moderate (6 studies) or moderate to high (4 studies) intensity, lasting 20 to 150 min in duration, and were held 5 to 2 times weekly for a total of 2 to 6 months. In addition, the group exercise interventions were combined with psychosocial support in 7 studies which involved either group support sessions, counseling, education, or individual coaching. The group exercise sessions were performed in supervised groups (12 studies) or teams (2 studies) at a hospital (5 studies), community (5 studies), or university-affiliated facility (4 studies). Supervision was generally provided by a kinesiologist, an exercise physiologist, or a physiotherapist. Detailed characteristics of the group exercise regimens are presented in Table 2 based on the FITT (frequency, intensity, time, and type) principle.

**Table 2:** Group exercise intervention characteristics

Study	Participants in each group session (***n***)	Type of group exercise (i.e., team or supervised group)	Frequency, days per week	Intervention period (mo)	Intensity	Time, session duration (min)	Type of exercise	Adherence (%)	Setting of exercise (i.e., home, community, hospital-based facility)	Co-interventions (i.e., educational, psychological, or psychosocial support)	Change from baseline in physical activity outcome compared to control
Alibhai et al. (2024) [19]	4–8	Supervised group	4–5	6	Moderate to vigorous intensity/60–70% of HRmax	60	Aerobic (modality determined by participant preference) and upper and lower body resistance training	52.9%	Hospital facility	None	6MWT, no statistically significant difference in the mean increase in 6MWT at 6 months between group and individual exercise, 31 m vs. 35 m, *p* = 0.86
Bjerre et al. (2019) [20]	10–14	Supervised team	2	6	NR	60	Football training	59%	Community facility	None	Median MET, no difference in the increase in median MET at 1 year between football training and usual care, 387 (IQR 3,539) vs. 589 (IQR 5,899), *p* value NR
Borsati et al. (2024) [21]	4–6	Supervised group	2	3	Moderate intensity/3–5 RPE	60	Aerobic (cycling or walking) and resistance training (upper and lower body with resistance bands)	100%	University-affiliated facility	None	6MWT, no statistically significant difference in the mean increase in 6MWT at 3 months between group and individual exercise, 30.7 m vs. 28.6 m vs. 20.5 m, *p* = 0.42
Cho et al. (2006) [22]	NR	Supervised group	3	2.5	Low to moderate intensity/40–60% of HRmax	90	NR	86%	Hospital facility	Home-based stretching, psychology-based education and peer support group activity	Shoulder joint range of motion, increased from 81.4 to 92.9% vs. 80.1 to 81.4% at 10 weeks with group exercise compared to usual care, *p* = < 0.0001
Desbiens et al. (2017) [23]	NR	Supervised group	2	3	Low to moderate intensity	50	Aerobic and strength training	38.5%	Hospital facility	None	6MWT, no difference in the median increase in 6MWT at 12 weeks between group and individual exercise, 516 to 590 m vs. 555 to 600 m,* p* > 0.05
Galvao et al. (2025) [24]	10–12	Supervised group	3	6	Moderate to vigorous intensity/60–85% of HRmax	30—20	Aerobic and resistance training	NR	University-affiliated facility	With or without psychosocial education	400 m walk, no difference in mean change from baseline in 400 m at 6 months between group exercise and usual care, −20.0 m, *p* = 0.13
Kim et al. (2020) [25]	3–6	Supervised group for 6 weeks and individual unsupervised for 6 weeks	1–2	3	Low, moderate, and high intensity	NR	Aerobic and strength training	90%	University-affiliated facility	Social capital activation strategy with 8 sessions of bonding social capital and preparing for bridging social capital	GPAQ, 1.5 times higher increase in metabolic equivalence of task post-intervention with group exercise compared to usual care, 2239.13 vs. 130.17, *p* = 0.046
Leach et al. (2019) [26]	3–10	Supervised group	2	2	Moderate to vigorous intensity/55–75% of HRmax	60	Aerobic (treadmill, elliptical, stationary bike, and outdoor walking) and strength training (upper and lower body)	87.9–70.1%	University-affiliated facility	Physical activity education and discussion sessions	MET-hours per week, no difference in mean change from baseline in MET-hours per week at 8 weeks between group and individual exercise, 21.7 vs. 21.3, *p* = 0.91
Mardani et al. (2020) [27]	5–8	Supervised group and individual unsupervised	1	2.5	Low to moderate intensity	150–60	Aerobic (walking), resistant (upper and lower body), flexibility (upper and lower body) and pelvic floor muscle training	80.09%	Community facility	None	Physical function domain of the EORTC QLQ C-30, increase in mean physical functioning score from baseline with group exercise compared to usual care, 60.19 to 72.57 vs. 55.74 to 60.19, *p* < 0.001
Møller et al. (2020) [28]	10–14	Supervised group	3	2.5	Low, moderate, and high intensity	90–60	Aerobic (stationary cycling) resistance training, relaxation training and massage	70.2%	Hospital facility	Health counselling and symptom guidance	CRF/VO_2_peak, no difference in decreased from baseline in CRF at 12 weeks and increased from 12 to 39 weeks between group and individual exercise, *p* < 0.001
Mutrie et al. (2007) [29]	NR	Supervised group	2	3	Moderate to vigorous intensity/50–75% of HRmax	45	Aerobic and strength training	NR	Community facility	None	12MWT, higher increase in the mean change from baseline in 12MWT with group exercise compared to usual care at 3 and 6 months, 129 m, and 105 m, *p* = < 0.0001
Naumann et al. (2012) [30]	NR	Supervised group	3	2.25	Moderate intensity	45–60	Aerobic (cycling, cross-training, brisk walking) and strength training, core training, hydrotherapy, and flexibility	74%	Community facility	Weekly group support with counseling	NR
Porserud et al. (2014) [31]	NR	Supervised group and self-directed individual walking	2	3	NR	45	Endurance (walking) and strength (balance and stretching) training	87–76%	Hospital facility	None	6MWT, higher increase in mean distance walked from baseline at 12 weeks and 1 year with group exercise compared to usual care, 112.9 m vs. 62.8 m, *p* = 0.013 and 23.8 m vs. −19.2 m, *p* = 0.010
Tjoe et al. (2020) [32]	NR	Supervised team and 3–4 times weekly individual home exercise	2	3.5	Moderate intensity/5–8 MET	90	Triathlon training (swimming, biking, and running) with aerobic and core exercises	NR	Community facility	Twice weekly coaching on lifestyle and exercise success strategies	6MWT, higher increase in mean distance walked from baseline at 14 weeks with triathlon training compared to usual care, 26 m vs. 4.8 m, *p* = 0.05

*6MWT* 6-min walk test, *12MWT* 12-min walk test, *CRF/VO*_*2*_*peak* cardiorespiratory fitness/peak oxygen update, *EORTC QLQ C-30* European Organization for Research and Treatment of Cancer Quality of Life and Core Questionnaire, *GPAQ* Global Physical Activity Questionnaire, *HRmax* heart rate maximum, *IPAQ* International Physical Activity Questionnaire, *MET* metabolic equivalent of task, *NR* not reported, *RPE* Borg Rating of the Perceived Exertion Scale

The effect of the group-based exercise intervention on physical functioning post-intervention was measured using the distanced walked during a 6-min walk test (6MWT) in 5 studies, metabolic equivalents of task (MET) in 2 studies, or the 12-min walk test (12MWT) in 1 study [19–21, 23, 26, 29, 31, 32]. Participants receiving the group-based intervention walked 129 to 22 m further than those receiving usual care in 3 studies [29, 31, 32]. Of the 8 studies that compared group exercise to usual care, only 2 studies noted no statistically significant improvement from baseline in physical functioning between the two groups after the intervention [20, 22, 24, 25, 27, 29, 31, 32]. For the 6 studies comparing group exercise to individual exercise, none showed higher improvements in physical functioning in the group-based exercise intervention compared to the individual exercise control [19, 21, 23, 26, 28, 30].

### Effect of group exercise on quality of life

The change from baseline in quality of life was quantified by changes in Functional Assessment of Cancer Therapy scores for breast (FACT-B) or prostate cancer (FACT-P) in 8 studies and European Organization for Research and Treatment of Cancer Quality of life and Core Questionnaire scores (EORTC QLQ C-30) in 3 studies [19–21, 23, 25–30, 32]. Group exercise was associated with a −0.7 to 1.9-point change from baseline in FACT-P scores, 8.1 to 32.85-point increase in FACT-B, and a −5 to −0.71-point changes in EORTC score from baseline, where the minimal clinically important difference is 6 to 10 points for the FACT-P, 7 to 8 points for the FACT-B, and 10 points for the EORTC QLQ C-30 [33–35]. However, among the 6 studies that used individual exercise as their control, only 1 study reported a statistically different improvement from baseline in quality-of-life scores in the participants receiving the group-based exercise intervention whereas 5 of the 6 studies noted no difference between the two arms [16, 18, 20, 22, 24, 26]. The study that showed a higher increase from baseline in overall and cancer specific quality of life scores with group exercise compared to individual exercise designed the group intervention with strategies to promote positive group cohesion and measured group dynamics using the Physical Activity Group Environment Questionnaire [26]. Among the 8 studies that included usual care as their control arm, 6 studies demonstrated that a group exercise intervention resulted in higher improvements in quality-of-life outcomes [20, 22, 24, 25, 27, 29, 31, 32]. Further details relating to the effect of group exercise on quality-of-life metrics are described in Table 3.

**Table 3:** Group exercise and patient-reported outcomes

Study	Quality-of-life survey	Effect of group exercise on quality-of-life survey scores compared to control	Fatigue survey	Effect of group exercise on fatigue survey scores compared to control	Depression survey	Effect of group exercise on depression survey scores compared to control	Sexual function survey	Effect of group exercise on sexual function survey scores compared to control
Alibhai et al. (2024) [19]	† FACT-P † FACT-G	No difference in the mean (SD) change from baseline in FACT-P scores at 6 months post-intervention between group and individual exercise, −0.7 (5.2) vs. −0.1 (6.6), *p* = 0.73 No difference in the mean (SD) change from baseline in FACT-G scores at 6 months post-intervention between group and individual exercise, −2.1 (7.8) vs. −2.8 (29.0),* p* = 0.92	‡ FACT-F	Lower decrease from baseline in mean (SD) FACT-F scores with group exercise compared to individual exercise at 6 months, 3.6 (6.1) vs. 12.1 (8.1), *p* = 0.040	NR	Not applicable	NR	Not applicable
Bjerre et al. (2019) [20]	† FACT-P † Mental Component Summary of Short Form-12	No difference in the mean change from baseline in FACT-P scores at 6 months post-intervention between football training and usual in the intention to treat and per protocol populations, 1.9, *p* = 0.325, 3.6,* p* = 0.119 Statistically significant increase in the mean change from baseline Mental Component Summary scores with football training compared to usual care at 1-year post-intervention, 2.9, *p* = 0.048	NR	Not applicable	NR	Not applicable	NR	Not applicable
Borsati et al. (2024) [21]	† EORTC QLQ C-30	No difference in the mean change from baseline in EORTC QLQ C-30 scores across all subscales between group and individual exercise at 3 months	‡‡ Fatigue subscale of EORTC QLQ C-30,	No difference in the mean increase from baseline in fatigue subscale scores between group and individual exercise at 3 months, 38.89 vs. 33.33 vs. 33.33, *p* = 0.608	NR	Not applicable	NR	Not applicable
Cho et al. (2006) [22]	† Chae and Choe instrument	Increase in the mean change from baseline in quality-of-life scores with group exercise compared to usual care, 0.9 vs. −0.1, *p* = 0.002	NR	Not applicable	NR	Not applicable	NR	Not applicable
Desbiens et al. (2017) [23]	† FACT-B † FACT-G	No difference in increase from baseline in median FACT-B scores at 12 weeks between group and individual exercise, 106 to 112 vs. 109 to 117,* p* > 0.05 No difference in increase from baseline in median FACT-G scores at 12 weeks between group and individual exercise, 82 to 87 vs. 90 to 94, *p* > 0.05	‡ FACT-F	No difference in increase from baseline in median FACT-F scores at 12 weeks, 115 to 130 vs. 133 to 137, *p* > 0.05	NR	Not applicable	NR	Not applicable
Galvao et al. (2025) [24]	NR	Not applicable	NR		NR	Not applicable	*IIEF	Increase in the mean change from baseline in mean IIEF scores at 6 months post-intervention compared to control, 5.1 vs. 1.0 points, *p* = 0.04
Kim et al. (2020) [25]	† FACT-B	No difference in increase from baseline in mean FACT-B scores post-intervention at 12 weeks between group and usual care, 32.85 vs. 28.40, *p* = 0.329	‡‡ R-PFS-K	No difference in decrease from baseline in mean fatigue scores post-intervention at 12 weeks between group and usual care, 1.34 vs. 0.48, *p* = 0.052	†† K-HADS	No difference in decrease from baseline in mean depression scores post-intervention at 12 weeks between group and usual care, 1.04 vs. 0.52, *p* = 0.488	NR	Not applicable
Leach et al. (2019) [26]	† FACT-B	Overall mean total FACT-B increased from baseline at 8 weeks post intervention with group exercise compared to individual exercise, 111.7 to 119.8 vs. 104.7 to 108.2, *p* = 0.041 Breast cancer–specific concerns of the FACT-B improved with group exercise compared to individual exercise, 26.3 to 29.2 vs. 24.8 to 25.3, *p* = 0.046	NR	Not applicable	NR	Not applicable	NR	Not applicable
Mardani et al. (2020) [27]	† EORTC QLQ C-30	No change from baseline in mean total EORTC QLQ C-30 scores post-intervention between group and usual care, 34.04 to 33.33 vs. 36.57 to 35.18, *p* = 0.41 Increase from baseline in mean role function domain of the EORTC QLQ C-30 scores post-intervention with group exercise compared to usual care, 58.57 to 70.95 vs. 59.72 to 61.57, *p* < 0.002 Increase from baseline in mean social function domain of the EORTC QLQ C-30 scores post-intervention with group exercise compared to usual care, 40.47 to 58.1 vs. 47.68 to 53.24, *p* < 0.001	‡‡ Fatigue subscale of EORTC QLQ C-30	Decrease from baseline in mean fatigue domain of the EORTC QLQ C-30 scores post-intervention with group exercise compared to usual care, 52.38 to 33.97 vs. 49.38 to 42.59, *p* < 0.001	NR	Not applicable	*Sexual activity and sexual function domain of EORTC QLQ C-30	Increase from baseline in mean sexual activity domain of the EORTC QLQ C-30 scores post-intervention with group exercise compared to usual care, 28.33 to 31.25 vs. 23.61 to 21.84, *p* = 0.001
Møller et al. (2020) [28]	† EORTC QLQ C-30	No difference in the decrease from baseline in mean total EORTC QLQ C-30 scores post-intervention at 12 weeks between group and individual exercise, 61 to 56 vs. 62 to 52, *p* = 0.128 No difference in the increase from baseline in mean total EORTC QLQ C-30 scores at 39 weeks between group and individual exercise, 62 to 76 vs. 63 to 69, *p* = 0.051	‡‡ Fatigue subscale of EORTC QLQ C-30	No difference in the increase in mean fatigue scores from baseline at 12 weeks between group and individual exercise, 42 to 58 vs. 48 to 59, *p* = 0.349 No difference in the decrease below baseline in mean fatigue scores at 39 weeks between group and individual exercise, 40 to 29 vs. 46 to 38, *p* = 0.43	†† HADS	No difference in the increase in mean depression scores from baseline at 12 weeks between group and individual exercise, 3.3 to 4.6 vs. 3.7 to 5.7, *p* = 0.625 No difference in the decrease in mean depression scores post-intervention at 39 weeks between group and individual exercise, 3.2 to 2.5 vs. 3.4 to 3.1, *p* = 0.519	NR	Not applicable
Mutrie et al. (2007) [29]	† FACT-B † FACT-G	Increase from baseline in the mean FACT-B scores at 3 months and at 6 months post intervention with group exercise compared to usual care, 2.5, *p* = 0.0007, and 1.5, *p* = 0.039 No statistically significant difference in change from baseline in mean FACT-G scores at 3 months and at 6 months post intervention between group exercise and usual care, 1.0, *p* = 0.60 and 3.6, *p* = 0.053	‡ FACT-F	No difference in the change from baseline in mean FACT-F scores at 3 months and at 6 months post-intervention between group exercise and usual care, 2.3, *p* = 0.091 and 1.9, *p* = 0.15	†† Beck Depression Inventory	No difference in the change from baseline in mean depression scores at 3 months and at 6 months post-intervention between group exercise and usual care, −1.7, *p* = 0.083 and −1.8 *p* = 0.064	NR	Not applicable
Naumann et al. (2012) [30]	† FACT-B	Smaller increase from baseline in total FACT-B scores (95% CI) post-intervention at 9 weeks with group exercise compared to individual exercise and larger increase from baseline with group exercise compared to usual care, 9.8 (4.1–15.6) vs. 14.9 (8.7–21.6) vs. 1.8 (−5.0 to 8.6), *p* < 0.023	NR	Not applicable	NR	Not applicable	NR	Not applicable
Porserud et al. (2014) [31]	† SF-36	Increase from baseline in role physical domain of the SF-36 at 12 weeks with group exercise compared to usual care, 100 vs. 37.5, *p* = 0.031 No difference in change from baseline in all other domains of the SF-36 at 12 weeks and 1 year between group exercise and usual care	NR	Not applicable	NR	Not applicable	NR	Not applicable
Tjoe et al. (2020) [32]	† FACT-B	Increase from baseline in mean total FACT-B scores at 14 weeks post intervention with group exercise compared to usual care, 114 to 125 vs. 113 to 115, *p* = 0.05	‡ FACIT-F	Increase from baseline in mean total FACIT-F scores at 14 weeks post intervention compared to control, 40 to 47 vs. 38 to 40, *p* = 0.04	NR	Not applicable	NR	Not applicable

*CI* confidence interval, *EORTC QLQ C-30* European Organization for Research and Treatment of Cancer Quality of Life and Core Questionnaire, *FACIT-F* Functional Assessment of Chronic Illness Therapy-Fatigue, *FACT-B* Functional Assessment of Cancer Therapy–Breast, *FACT-F* Functional Assessment of Cancer Therapy–Fatigue, *FACT-P* Functional Assessment of Cancer Therapy–Prostate, *FACT-G* Functional Assessment of Cancer Therapy–General, *IIEF* International Index of Erectile Function, *K-HADS* Korean version of the Hospital Anxiety and Depression Scale, *NR* not reported, *R-PFS-K* Korean-Revised Piper Fatigue Scale, *SD *standard deviation, *SF-36*, Short Form 36

^†^Higher scores represent better quality of life

^‡^Higher scores represent less fatigue

^‡‡^Higher scores represent more fatigue

^††^Higher scores represent worse depression

*Higher scores represent improved sexual function

### Effect of group exercise on secondary patient-reported outcomes

Other patient-reported outcome measures included fatigue, depression, and sexual function. Of the 8 studies that measured patient-reported fatigue, 3 studies used the FACT-F, 3 studies used the fatigue subscale of the EORTC QLQ C-30, 1 study used the Functional Assessment of Chronic Illness Therapy-Fatigue (FACIT-F), and 1 study used the Korean-Revised Piper Fatigue Scale (R-PFS-K) [19, 21, 23, 25, 27–29, 32]. The minimal important difference for the FACT-F, fatigue subscale of the EORTC QLQ C-30, and FACIT-F is 3 points, 2.2 to 4.8 points, and 3 to 4 points, respectively [36–38]. Alibhai et al. reported a smaller 3.6-point decrease in FACT-F scores for those participating in group exercise compared to individual exercise (*p* = 0.04) while the two other studies showed no difference between group exercise and usual care nor individual exercise [19, 23, 29]. One study showed a statistically significant 18.41-point improvement from baseline in the fatigue subscale scores of the EORTC QLQ C-30 in the group-based exercise intervention arm compared to usual care (*p* < 0.001) while another study that used this subscale showed no significant difference in the changes between group exercise and individual exercise (*p* = 0.349) [27, 28]. One study showed a 7-point improvement in FACIT-F scores compared to usual care (*p* = 0.04) [32].

Three studies measured changes from baseline in depression quantified by the HADS, K-HADS, and Beck Depression Inventory scales [25, 28, 29]. Kim and Moller et al. reported a 1.04 and 0.7-point decrease in the K-HADS and HADS scores, respectively, in participants receiving a group-based exercise intervention; these changes in depression metrics were not statistically different from control groups [25, 28]. Mutrie et al. showed no improvement in depression scores after the group-based exercise intervention [29].

Two studies measured change from baseline in sexual function using the sexual function/activity subscales of the EORTC QLQ C-30 and the International Index of Erectile Function (IIEF) [24, 27]. Both of those studies reported an improvement in sexual function scores after a group-exercise intervention compared to usual care. Galvao et al. showed a statistically significant 5.1-point increase in IIEF scores (*p* = 0.04) where 4 points is the minimum important difference for the IIEF [24, 39]. Mardani et al. reported a 2.92-point increase (*p* = 0.001) in the sexual function/activity subscales of the EORTC QLQ C-30 [27].

## Discussion

To our knowledge, this is the first comprehensive study examining the use of group-based exercise interventions in patients with a urologic, gynecologic, or breast malignancy. We report that group-based exercise can lead to clinically meaningful improvements in patient-reported outcomes such as quality of life, fatigue, and sexual function compared to usual care. However, these improvements were not significantly different when compared to individual-based exercise. It is well reported that patients with these malignancies benefit from exercise and require supportive measures to improve their quality of life [7–9]. However, we were unable to demonstrate an overt added supportive benefit of group-based exercise over individual exercise.

In our review, only one study measured the level of group cohesion and the quality of the group dynamic that resulted from the group exercise intervention [26]. It is possible that unmeasured group dynamic quality could account for variations in the impact of group exercise compared to individual exercise among the included studies on quality-of-life metrics. Poor or absent group dynamics may translate to group exercise participants experiencing no difference in their sense of community and ultimately only garnering the quality-of-life benefits relating to exercise alone in comparison to individual exercise participants. Conversely, it is possible that the improvements in patient-reported metrics with group exercise are solely due to the presence of a supportive group dynamic. It is well reported that a supportive group dynamic founded in shared experiences builds camaraderie and trust among cancer patients which can positively impact social quality of life [15, 40]. However, whether this effect is additive when combined with exercise could not be determined in our study.

A prior 2009 meta-analysis investigating the potential advantages of group-based exercise over individual exercise showed no additional benefit of group exercise on quality of life in breast cancer patients. Similar to our findings, the authors of that study noted that studies reporting no improvement in social quality of life did not include data on group dynamics [41]. Thus, our study confirms the importance of examining group dynamic and cohesion quality to appropriately assess the impact of a group-based exercise intervention on social functioning; group dynamic quality serves as an important metric for determining the effectiveness of a group-based intervention in providing social support [42, 43]. In addition, group cohesion can have important implications for long-term exercise adherence and the development of self-efficacy after a group exercise intervention [43].

The current review provides new information on the use of group exercise in prostate and bladder cancer patients given that the majority of the literature in this realm includes investigations in breast cancer patients. In addition, we highlight gaps in the use of group exercise for gynecologic malignancies such as uterine, endometrial, and cervical cancer as these cancer types are understudied in exercise research [44, 45].

To build on this work, future studies will involve examining the mechanisms involved in developing positive group cohesion and integrating strategies that promote a supportive group dynamic into group exercise interventions. In this way, group dynamics will serve as a potential instrument and component of group exercise interventions to improve social support for cancer patients with urologic, gynecologic, and breast malignancies.

The primary strength of this study is its inclusion of patients with different cancer types but similar quality of life challenges which allowed for a large sample size. However, due to the absence of individual-level data, a formal meta-analysis was not possible. In addition, while participants with different cancer types were included, the study population only consisted of prostate, breast, and bladder cancer patients which limits its generalizability to other urologic and gynecologic cancer types. Lastly, while our study included patients who received a group-based exercise intervention, 6 of the 14 included studies did not report the number of participants in each group exercise session. Thus, it is uncertain whether those studies represent a true group-based intervention.

## Conclusion

We evaluate the impact of group-based exercise interventions on patient-reported quality of life among individuals with breast, prostate, or bladder cancer compared to individual exercise and usual care. In the absence of measured group cohesion, we were unable to demonstrate a clear advantage of group-based exercise over individual exercise. These findings highlight the importance of assessing positive group dynamics as a key metric of the supportive value of group-based exercise intervention.

## Supplementary Information

Below is the link to the electronic supplementary material.

ESM 1 (DOCX 93.2 KB)

## Data Availability

No datasets were generated or analysed during the current study.
