# Supplementary material for: Group exercise interventions and their impact on patient-reported outcomes in urologic, gynecologic, and breast cancers
Source: Support Care Cancer. 2026 Nov 12;34(12):1219. doi: 10.1007/s00520-026-11447-7 (PMC13759640; doi:10.1007/s00520-026-11447-7)
Supplement: Supplementary file 1 [file 520_2026_11447_MOESM1_ESM.docx]

**Supplemental Information: Group Exercise Interventions and Their Impact on Patient-Reported Outcomes in Urologic, Gynecologic and Breast Cancers**

**Supplemental Text 1. Literature Search Strategies**
conducted on December 15, 2025

**PubMed via Medline**

(("group exercise"[tiab:~2] OR "group exercises"[tiab:~2] OR "groups exercise"[tiab:~2] OR "groups exercises"[tiab:~2] OR "group exercising"[tiab:~2] OR "groups exercising"[tiab:~2] OR "group fitness"[tiab] OR "instructor-led fitness"[tiab:~2] OR "instructor-led exercis*"[tiab] OR "community-based fitness"[tiab] OR "community-based exercise*"[tiab] OR "group physical activit*"[tiab] OR "group strength training"[tiab] OR "instructor-led strength training"[tiab:~2] OR "walking groups"[tiab:~2] OR "group walks"[tiab:~2] OR "group aerobic*"[tiab] OR "exercise class*"[tiab] OR "fitness class*"[tiab]) OR ((Exercise[mesh] OR "Physical Fitness"[mesh] OR "Exercise Therapy"[mesh]) AND (group[ti] OR "instructor-led"[ti] OR "community-based"[ti]))) AND ("urologic cancer*"[tiab] OR "urologic neoplasm*"[tiab] OR "urological cancer*"[tiab] OR "urological neoplasm*"[tiab] OR "urinary tract neoplasm" OR "cancer urinary"[tiab:~2] OR "bladder cancer*"[tiab] OR "prostate cancer*"[tiab] OR "kidney cancer*"[tiab] OR "cancer kidneys"[tiab:~2] OR "testicular cancer*"[tiab] OR "bladder neoplasm*"[tiab] OR "prostate neoplasm*"[tiab] OR "kidney neoplasm*"[tiab] OR "neoplasm kidney"[tiab:~2] OR "renal cell carcinoma*"[tiab] OR "testicular neoplasm*"[tiab] OR “urethral cancer*”[tiab] OR “urethral neoplasm*”[tiab] OR “urethra cancer”[tiab:~2] OR “urethra neoplasm”[tiab:~2] OR “penile cancer*”[tiab] OR “penile neoplasm*”[tiab] OR “penis cancer”[tiab:~2] OR “penis neoplasm”[tiab:~2] OR “urogenital cancer*”[tiab] OR “urogenital neoplasm*”[tiab] OR "Urologic Neoplasms"[Mesh] OR "breast cancer"[tiab:~2] OR "breast neoplasm"[tiab:~2] OR "breast carcinoma"[tiab:~2] OR "breast cancers"[tiab:~2] OR "breast neoplasms"[tiab:~2] OR "breast carcinomas"[tiab:~2] OR "uterine cancer*"[tiab] OR "uterine neoplasm*"[tiab] OR "uterus cancer"[tiab:~2] OR "uterus neoplasm"[tiab:~2] OR "cervical cancer*"[tiab] OR "cervical neoplasm*"[tiab] OR "cancer cervix"[tiab:~2] OR "ovarian cancer*"[tiab] OR "ovarian neoplasm*"[tiab] OR "cancer ovary"[tiab:~2] OR "cancer ovaries"[tiab:~2] OR "vaginal cancer*"[tiab] OR "vaginal neoplasm*"[tiab] OR "cancer vagina"[tiab:~2] OR "neoplasm vagina"[tiab:~2] OR "vulvar cancer*"[tiab] OR "vulvar neoplasm*"[tiab] OR "cancer vulva"[tiab:~2] OR "neoplasm vulva"[tiab:~2] OR "endometrial cancer*"[tiab] OR "endometrial neoplasm*"[tiab] OR "Breast Neoplasms"[mesh] OR "Genital Neoplasms, Female"[Mesh]) NOT ((infant[mesh] OR child[mesh] OR adolescent[mesh]) NOT adult[mesh]) NOT (letter[pt] OR comment[pt] OR editorial[pt] OR "case reports"[pt]) AND english[la] AND 2005 : 3000[dp]

**Embase via Elsevier**

((('group*' NEAR/3 'exercis*'):ti,ab,kw) OR 'group fitness':ti,ab,kw OR (('instructor-led' NEAR/3 'fitness'):ti,ab,kw) OR 'instructor-led exercis*':ti,ab,kw OR 'community-based fitness':ti,ab,kw OR 'community-based exercis*':ti,ab,kw OR 'group physical activit*':ti,ab,kw OR 'group strength training':ti,ab,kw OR (('instructor-led' NEAR/3 'strength' NEAR/3 'training'):ti,ab,kw) OR (('walk*' NEAR/3 'group*'):ti,ab,kw) OR 'group* near/3 aerobic*':ti,ab,kw OR 'exercise class*':ti,ab,kw OR 'fitness class*':ti,ab,kw OR 'exercise'/exp/mj OR 'fitness'/exp/mj) AND ('group':ti OR 'instructor-led':ti OR 'community-based':ti) AND (((urologic* NEAR/3 cancer*):ti,ab,kw) OR ((urologic* NEAR/3 neoplasm*):ti,ab,kw) OR ((urinary NEAR/3 tract NEAR/3 neoplasm*):ti,ab,kw) OR ((cancer* NEAR/3 urinary):ti,ab,kw) OR ((bladder NEAR/3 cancer*):ti,ab,kw) OR ((prostat* NEAR/3 cancer):ti,ab,kw) OR ((cancer* NEAR/3 kidney*):ti,ab,kw) OR ((testicular NEAR/3 cancer*):ti,ab,kw) OR ((testis NEAR/3 cancer):ti,ab,kw) OR ((bladder NEAR/3 neoplasm*):ti,ab,kw) OR ((prostat* NEAR/3 neoplasm*):ti,ab,kw) OR ((neoplasm* NEAR/3 kidney*):ti,ab,kw) OR ((renal NEAR/3 cell NEAR/3 carcinoma*):ti,ab,kw) OR ((testic* NEAR/3 neoplasm*):ti,ab,kw) OR ((penis NEAR/3 cancer*):ti,ab,kw) OR ((penile NEAR/3 cancer*):ti,ab,kw) OR ((urethr* NEAR/3 cancer*):ti,ab,kw) OR ((urethr* NEAR/3 neoplasm*):ti,ab,kw) OR 'urinary tract tumor'/exp/mj OR 'urogenital tract tumor'/exp/mj OR (('breast' NEAR/3 'cancer'):ti,ab,kw) OR (('breast' NEAR/3 'neoplasm'):ti,ab,kw) OR (('breast' NEAR/3 'carcinoma'):ti,ab,kw) OR (('breast' NEAR/3 'cancers'):ti,ab,kw) OR (('breast' NEAR/3 'neoplasms'):ti,ab,kw) OR (('breast' NEAR/3 'carcinomas'):ti,ab,kw) OR 'uterine cancer*':ti,ab,kw OR 'uterine neoplasm*':ti,ab,kw OR (('uterus' NEAR/3 'cancer'):ti,ab,kw) OR (('uterus' NEAR/3 'neoplasm'):ti,ab,kw) OR 'cervical cancer*':ti,ab,kw OR 'cervical neoplasm*':ti,ab,kw OR (('cancer' NEAR/3 'cervix'):ti,ab,kw) OR 'ovarian cancer*':ti,ab,kw OR 'ovarian neoplasm*':ti,ab,kw OR (('cancer' NEAR/3 'ovary'):ti,ab,kw) OR (('cancer' NEAR/3 'ovaries'):ti,ab,kw) OR 'vaginal cancer*':ti,ab,kw OR 'vaginal neoplasm*':ti,ab,kw OR (('cancer' NEAR/3 'vagina'):ti,ab,kw) OR (('neoplasm' NEAR/3 'vagina'):ti,ab,kw) OR 'vulvar cancer*':ti,ab,kw OR 'vulvar neoplasm*':ti,ab,kw OR (('cancer' NEAR/3 'vulva'):ti,ab,kw) OR (('neoplasm' NEAR/3 'vulva'):ti,ab,kw) OR 'endometrial cancer*':ti,ab,kw OR 'endometrial neoplasm*':ti,ab,kw OR 'breast cancer'/exp/mj OR 'female genital tract cancer'/exp/mj) NOT (([infant]/lim OR [child]/lim OR [adolescent]/lim) NOT [adult]/lim) AND [english]/lim AND [2005-01-01 to 2025-12-12]/ld

**CINAHL Complete via EBSCOHost**

**Advanced Search, Search Options:** Proximity Search, Filters: English, Publication Date 2005-2025, Adult, Academic Journals

((XB ((urologic* N2 cancer*) OR (urologic* N2 neoplasm*) OR (urinary N2 tract N2 neoplasm*) OR (cancer* N2 urinary) OR (bladder N2 cancer*) OR (prostat* N2 cancer) OR (cancer* N2 kidney*) OR (testicular N2 cancer*) OR (testis N2 cancer) OR (bladder N2 neoplasm*) OR (prostat* N2 neoplasm*) OR (neoplasm* N2 kidney*) OR (renal N2 cell N2 carcinoma*) OR (testic* N2 neoplasm*) OR (penis N2 cancer*) OR (penile N2 cancer*) OR (urethr* N2 cancer*) OR (urethr* N2 neoplasm*) OR (breast N2 cancer) OR (breast N2 neoplasm) OR (breast N2 carcinoma) OR (breast N2 cancers) OR (breast N2 neoplasms) OR (breast N2 carcinomas) OR “uterine cancer*” OR “uterine neoplasm*” OR (uterus N2 cancer) OR (uterus N2 neoplasm) OR “cervical cancer*” OR “cervical neoplasm*” OR (cancer N2 cervix) OR ovarian cancer* OR ovarian neoplasm* OR (cancer N2 ovary) OR (cancer N2 ovaries) OR “vaginal cancer*” OR “vaginal neoplasm*” OR (cancer N2 vagina) OR (neoplasm N2 vagina) OR “vulvar cancer*” OR “vulvar neoplasm*” OR (cancer N2 vulva) OR (neoplasm N2 vulva) OR “endometrial cancer*” OR “endometrial neoplasm*”) OR (MH "Urogenital Neoplasms+" OR MH "Breast Neoplasms+" OR MH "Genital Neoplasms, Female+")) AND ( XB ((group* N2 exercis*) OR “group fitness” OR “instructor-led fitness” OR “instructor-led exercis*” OR “community-based fitness” OR “community-based exercis*” OR “group physical activit*” OR “group strength training” OR (instructor-led N2 strength N2 training) OR (walk* N2 group*) OR (group* N2 aerobic*) OR “exercise class*” OR “fitness class*”))

**SportDiscus with Full Text via EBSCOHost**

**Advanced Search, Search Options:** Proximity Search, Filters: English, Publication Date 2005-2025, Academic Journals

XB ((group* N2 exercis*) OR “group fitness” OR “instructor-led fitness” OR “instructor-led exercis*” OR “community-based fitness” OR “community-based exercis*” OR “group physical activit*” OR “group strength training” OR (instructor-led N2 strength N2 training) OR (walk* N2 group*) OR (group* N2 aerobic*) OR “exercise class*” OR “fitness class*”) AND XB ((urologic* N2 cancer*) OR (urologic* N2 neoplasm*) OR (urinary N2 tract N2 neoplasm*) OR (cancer* N2 urinary) OR (bladder N2 cancer*) OR (prostat* N2 cancer) OR (cancer* N2 kidney*) OR (testicular N2 cancer*) OR (testis N2 cancer) OR (bladder N2 neoplasm*) OR (prostat* N2 neoplasm*) OR (neoplasm* N2 kidney*) OR (renal N2 cell N2 carcinoma*) OR (testic* N2 neoplasm*) OR (penis N2 cancer*) OR (penile N2 cancer*) OR (urethr* N2 cancer*) OR (urethr* N2 neoplasm*) OR (breast N2 cancer) OR (breast N2 neoplasm) OR (breast N2 carcinoma) OR (breast N2 cancers) OR (breast N2 neoplasms) OR (breast N2 carcinomas) OR “uterine cancer*” OR “uterine neoplasm*” OR (uterus N2 cancer) OR (uterus N2 neoplasm) OR “cervical cancer*” OR “cervical neoplasm*” OR (cancer N2 cervix) OR ovarian cancer* OR ovarian neoplasm* OR (cancer N2 ovary) OR (cancer N2 ovaries) OR “vaginal cancer*” OR “vaginal neoplasm*” OR (cancer N2 vagina) OR (neoplasm N2 vagina) OR “vulvar cancer*” OR “vulvar neoplasm*” OR (cancer N2 vulva) OR (neoplasm N2 vulva) OR “endometrial cancer*” OR “endometrial neoplasm*”)

**Web of Science Core Collection**

**Search Terms in Title or Abstract via Advanced Search; Exact Search**

“group exercise” OR “group fitness” OR (instructor-led NEAR/3 fitness) OR instructor-led exercis* OR community-based fitness OR community-based exercis* OR group physical activit* OR group strength training OR (instructor-led NEAR/3 strength NEAR/3 training) OR (walk* NEAR/3 group*) OR (group* NEAR/3 aerobic*) OR “exercise class*” OR “fitness class*”

AND

(urologic* NEAR/3 cancer*) OR (urologic* NEAR/3 neoplasm*) OR (urinary NEAR/3 tract NEAR/3 neoplasm*) OR (cancer* NEAR/3 urinary) OR (bladder NEAR/3 cancer*) OR (prostat* NEAR/3 cancer) OR (cancer* NEAR/3 kidney*) OR (testicular NEAR/3 cancer*) OR (testis NEAR/3 cancer) OR (bladder NEAR/3 neoplasm*) OR (prostat* NEAR/3 neoplasm*) OR (neoplasm* NEAR/3 kidney*) OR (renal NEAR/3 cell NEAR/3 carcinoma*) OR (testic* NEAR/3 neoplasm*) OR (penis NEAR/3 cancer*) OR (penile NEAR/3 cancer*) OR (urethr* NEAR/3 cancer*) OR (urethr* NEAR/3 neoplasm*) OR (breast NEAR/3 cancer) OR (breast NEAR/3 neoplasm) OR (breast NEAR/3 carcinoma) OR (breast NEAR/3 cancers) OR (breast NEAR/3 neoplasms) OR (breast NEAR/3 carcinomas) OR “uterine cancer*” OR “uterine neoplasm*” OR (uterus NEAR/3 cancer) OR (uterus NEAR/3 neoplasm) OR “cervical cancer*” OR “cervical neoplasm*” OR (cancer NEAR/3 cervix) OR “ovarian cancer*” OR “ovarian neoplasm*” OR (cancer NEAR/3 ovary) OR (cancer NEAR/3 ovaries) OR “vaginal cancer*” OR “vaginal neoplasm*” OR (cancer NEAR/3 vagina) OR (neoplasm NEAR/3 vagina) OR “vulvar cancer*” OR “vulvar neoplasm*” OR (cancer NEAR/3 vulva) OR (neoplasm NEAR/3 vulva) OR “endometrial cancer*” OR “endometrial neoplasm*”

**Cochrane Database of Systematic Reviews**

**Search Terms in Title/Abstract/Keyword via Advanced Search**

“group exercise” OR “group fitness” OR (instructor-led NEAR/3 fitness) OR "instructor-led exercise" OR "community-based fitness" OR "community-based exercise" OR "group physical activities" OR "group strength training" OR (instructor-led NEAR/3 strength NEAR/3 training) OR (walk* NEAR/3 group*) OR (group* NEAR/3 aerobic*) OR (exercise NEXT class*) OR (fitness NEXT class*)

(urologic* NEAR/3 cancer*) OR (urologic* NEAR/3 neoplasm*) OR (urinary NEAR/3 tract NEAR/3 neoplasm*) OR (cancer* NEAR/3 urinary) OR (bladder NEAR/3 cancer*) OR (prostat* NEAR/3 cancer) OR (cancer* NEAR/3 kidney*) OR (testicular NEAR/3 cancer*) OR (testis NEAR/3 cancer) OR (bladder NEAR/3 neoplasm*) OR (prostat* NEAR/3 neoplasm*) OR (neoplasm* NEAR/3 kidney*) OR (renal NEAR/3 cell NEAR/3 carcinoma*) OR (testic* NEAR/3 neoplasm*) OR (penis NEAR/3 cancer*) OR (penile NEAR/3 cancer*) OR (urethr* NEAR/3 cancer*) OR (urethr* NEAR/3 neoplasm*) OR (breast NEAR/3 cancer) OR (breast NEAR/3 neoplasm) OR (breast NEAR/3 carcinoma) OR (breast NEAR/3 cancers) OR (breast NEAR/3 neoplasms) OR (breast NEAR/3 carcinomas) OR (uterine NEAR/3 cancer*) OR (uterine NEAR/3 neoplasm) OR (uterus NEAR/3 cancer*) OR (uterus NEAR/3 neoplasm*) OR (cervical NEAR/3 cancer*) OR (cervical NEAR/3 neoplasm*) OR (cancer NEAR/3 cervix) OR (ovarian NEAR/3 cancer*) OR (ovarian NEAR/3 neoplasm*) OR (cancer NEAR/3 ovary) OR (cancer NEAR/3 ovaries) OR (vaginal NEAR/3 cancer*) OR (vaginal NEAR/3 neoplasm*) OR (cancer NEAR/3 vagina) OR (neoplasm NEAR/3 vagina) OR (vulvar NEAR/3 cancer*) OR (vulvar NEAR/3 neoplasm*) OR (cancer NEAR/3 vulva) OR (neoplasm NEAR/3 vulva) OR (endometrial NEAR/3 cancer*) OR (endometrial NEAR/3 neoplasm*)

**Supplemental Table 1. Risk-of-Bias**

|  | **D1** | **D2** | **D3** | **D4** | **D5** | **Overall risk of bias** |  |
| --- | --- | --- | --- | --- | --- | --- | --- |
| Alibhai et al. 2024 |  |  |  |  |  |  | D1: Domain 1, Risk of bias arising from the randomization process  D2: Domain 2, Risk of bias due to deviations from the intended interventions (effect of assignment to intervention)  D3: Domain 3, Risk of bias from missing outcome data  D4: Domain 4, Risk of bias in measurement of the outcome  D5: Domain 5, Risk of bias in selection of the reported result  Low Risk  Some concern  High Risk |
| Bjerre et al. 2019 |  |  |  |  |  |  |  |
| Borsati et al. 2024 |  |  |  |  |  |  |  |
| Cho et al. 2006 |  |  |  |  |  |  |  |
| Desbiens et al. 2017 |  |  |  |  |  |  |  |
| Galvao et al. 2025 |  |  |  |  |  |  |  |
| Kim et al. 2020 |  |  |  |  |  |  |  |
| Leach et al. 2019 |  |  |  |  |  |  |  |
| Mardani et al. 2020 |  |  |  |  |  |  |  |
| Møller et al. 2020 |  |  |  |  |  |  |  |
| Mutrie et al. 2007 |  |  |  |  |  |  |  |
| Naumann et al. 2012 |  |  |  |  |  |  |  |
| Porserud et al. 2014 |  |  |  |  |  |  |  |
| Tjoe et al. 2020 |  |  |  |  |  |  |  |
